# NMR Methodology for Measuring Dissolved O_2_ and Transport in Lithium–Air Batteries

**DOI:** 10.1021/acs.jpcc.3c00991

**Published:** 2023-05-22

**Authors:** Evelyna Wang, Erlendur Jónsson, Clare P. Grey

**Affiliations:** Yusuf Hamied Department of Chemistry, University of Cambridge, Lensfield Road, Cambridge CB2 1EW, U.K.

## Abstract

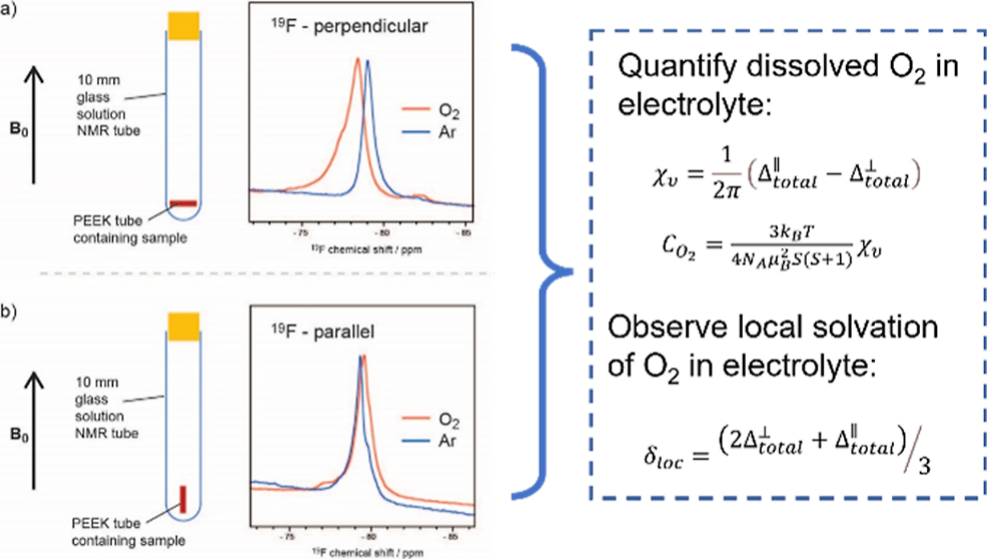

Similar to fuel cells, poor mass transport of redox active species, such as dissolved oxygen gas, is one of the challenges faced by lithium–air batteries (LABs). Capitalizing on the paramagnetic properties of O_2_, we used nuclear magnetic resonance (NMR) spectroscopy to measure oxygen concentration and transport in LAB electrolytes. Lithium bis(trifluoromethane) sulfonylimide (LiTFSI) in glymes or dimethyl sulfoxide (DMSO) solvents were investigated with ^1^H, ^13^C, ^7^Li, and ^19^F NMR spectroscopy, with the results showing that both the ^1^H, ^13^C, ^7^Li, and ^19^F bulk magnetic susceptibility shifts and the change in ^19^F relaxation times were accurate measures of dissolved O_2_ concentration. O_2_ saturation concentrations and diffusion coefficients were extracted that are comparable to values measured by electrochemical or pressure methods reported in the literature, highlighting the validity of this new methodology. This method also provides experimental evidence of the local O_2_ solvation environment, with results again comparable to previous literature and supported by our molecular dynamics simulations. A preliminary in situ application of our NMR methodology is demonstrated by measuring O_2_ evolution during LAB charging using LiTFSI in the glyme electrolyte. While the in situ LAB cell showed poor coulombic efficiency, since no additives were used, the O_2_ evolution was successfully quantified. Our work demonstrates the first usage of this NMR methodology to quantify O_2_ in LAB electrolytes, experimentally demonstrate solvation environments of O_2_, and detect O_2_ evolution in situ in a LAB flow cell.

## Introduction

Lithium–air batteries (LABs) promise extremely high energy densities.^1,2^ During discharge, lithium metal is oxidized at the anode to form Li^+^ ions and electrons, the latter traveling through an external circuit to reach the cathode and the former migrating through the electrolyte to the cathode. At the cathode, typically a porous carbon material, O_2_ is reduced and subsequently reacts with Li^+^ ions to form solid-phase Li_2_O_2_ discharge products, which deposit onto the surface of the cathode.^3^ The O_2_ from the environment must diffuse from the gas phase to the 3-phase boundary (solid electrode, liquid electrolyte, gaseous atmosphere) or dissolve into the electrolyte and again react at the electrode surface.^4,5^ Early work by Read et al. correlated the discharge capacity (and rate capability) in LABs with the O_2_ solubility in the electrolyte.^6−8^ Several studies have since endeavored to measure oxygen solubility in electrolytes; typical methods used include pressure and gas uptake experiments or electrochemical methods such as a rotating ring-disk electrode (RRDE).^6,9−12^ In pressure measurements, the gas overhead pressure above an electrolyte reservoir is monitored as a function of time. In general, a decrease in O_2_ solubility and diffusivity is observed for electrolytes with increasing viscosity, for example, by increasing the molecular weight of the glyme solvent used. Molecular dynamics (MD) simulations have also been employed to calculate O_2_ solubility and diffusivity, largely agreeing with experimental measurement trends.^10,11^

In this work, we employ a nuclear magnetic resonance (NMR) method to measure oxygen in LAB electrolytes. Paramagnetic species, such as radicals or (triplet) O_2_, in solution, can induce shifts in the measured NMR resonances as well as increase nuclear spin relaxation rates.^13,14^ The induced shifts in the resonance frequencies are caused by either hyperfine or bulk magnetic susceptibility (BMS) effects. The hyperfine interactions, sometimes termed a local effect, arise due to an interaction between the nucleus of interest and the unpaired electron spins on the paramagnetic species. This interaction can be of two types: contact or pseudo-contact. The first is caused by electron spin delocalization onto, or spin polarization of, the s-orbitals of the nucleus under observation, while the latter involves a through-space dipolar coupling between the electron and nuclear spins. In contrast, the BMS effect is a bulk response that results from the bulk susceptibility of the paramagnetic medium—be it a liquid, gas, or an array of particles. The presence of the paramagnetic species adds to the applied magnetic field felt by the nucleus and affects all nuclei at the same location by the same amount.

Our aim in this work was to explore the effects of paramagnetic O_2_ in LAB electrolytes using NMR spectroscopy, to establish whether either BMS or hyperfine effects can be used to quantify O_2_ concentrations and to explore O_2_ solvation. We start by briefly outlining the relevant theory for BMS shifts. We then compare the hyperfine shifts measured for various nuclei within our electrolytes, namely, various concentrations of lithium bis(trifluoromethane) sulfonylimide (LiTFSI) in glymes or dimethylsulfoxide (DMSO). We measure ^19^F, ^7^Li, and ^1^H solution NMR spectra and, using this theory, obtain information on O_2_ solvation within the electrolyte. We find that O_2_ appears to associate more closely with the F atoms of the LiTFSI salt. O_2_ dissolution from the gas phase into a liquid electrolyte was also measured using ^7^Li NMR spectroscopy, and the O_2_ diffusion constants were extracted. Finally, NMR relaxometry measurements were used to monitor O_2_ evolution during charge in a Li–air flow battery. To the best of our knowledge, these NMR methodologies have not been applied to measure paramagnetic O_2_ in LAB electrolytes before. The paper also describes the first experimental results on dissolved O_2_ solvation in LAB electrolytes. Paired with our flow-NMR setup, these methods enable direct observation of dissolved O_2_ during LAB operation.

## Methods

### Materials

LiTFSI was obtained from Sigma-Aldrich. Prior to use, LiTFSI salt was dried under a vacuum at 120 °C for 12 h. Diethylene glycol dimethyl ether (diglyme), triethylene glycol dimethyl ether (triglyme), tetraethylene glycol dimethyl ether (tetraglyme), and DMSO were purchased from Sigma-Aldrich. Diglyme, triglyme, and tetraglyme were refluxed under Ar with sodium metal, then distilled, and finally stored in an Ar-filled glovebox over molecular sieves. DMSO was used as is.

### Solution NMR

Electrolyte mixtures were prepared in the glovebox. For NMR measurements of the electrolytes under Ar, 0.3 mL of electrolyte was loaded into a 5 mm medium-walled glass solution NMR tube in the glovebox and sealed with a tap. For NMR measurements of oxygen-saturated electrolytes, O_2_ gas was bubbled into the electrolyte for 3 min with a syringe needle. A sealed capillary with deuterated benzene (C_6_D_6_) was added as a reference.

For orientation-dependent measurements, 7 mm sections of polyether ether ketone (PEEK) tubing, 1/16″ outer diameter, were used to hold electrolyte, ∼75 μL, and sealed with epoxy. The PEEK tubing with the electrolyte sample was then placed into a 10 mm solution NMR tube either horizontally or vertically and loaded into the probe.

NMR spectra were measured using a Bruker 300 MHz Advance III Spectrometer, with a magnetic resonance imaging (MRI) probe, using a single 90° pulse excitation for the different nuclei. Pulse lengths were 18.5 μs for ^19^F, 15 μs for ^7^Li, and 21 μs for ^1^H. Longitudinal *T*_1_ relaxation measurements were performed using a saturation recovery experiment, and transverse *T*_2_ relaxation measurements were performed using a Carr–Purcell–Meiboom–Gill (CPMG) pulse sequence.

### Flow Cell

A Li–air flow cell was set up following specifications from Milshtein et al.;^15^ however, instead of flowing both anolyte and catholyte, only one electrolyte was flown, and a polytetrafluoroethylene (PTFE) seal was placed on the Li metal anode side. A stainless-steel current collector was used with a Li metal anode, and a graphite current collector with etched interdigitated flow fields was used with the carbon electrode. Carbon electrodes, Sigracet 39BC, comprising carbon fiber paper coated with microporous carbon, were purchased from Fuel Cell Store and cut into 1 cm^2^ squares. Borosilicate glass fiber (GF) separators from Whatman were used. Lithium metal, 99.5%, was purchased from LTS Research and stored in an Ar-filled glovebox. The 0.25 M LiTFSI in the diglyme electrolyte was used. The total electrolyte volume was 20 mL.

Perfluoroalkoxy (PFA) tubing, 1/16 in. outer diameter, was used for most connections, and MasterFlex tubing 77202-60 #14 Chem Bio was used with the peristaltic pump. The electrolyte flow rate was ∼0.06 mL s^–1^. The LAB cell was initially assembled in the glovebox, where all components were sealed, before being placed onto a lab bench with the pump and Biologic SP-150 potentiostat. The electrolyte reservoir was then bubbled with O_2_ gas for 15 min. The cell was subjected to a 2.5 mA h discharge at a rate of 0.05 mA cm^–2^ to form Li_2_O_2_ at the carbon cathode. Subsequently, the electrolyte was purged with N_2_ to remove the O_2_ in the electrolyte. The PFA tubing was then connected to the NMR spectrometer to enable in situ measurement. The online flow-through design is described in previous reports.^16^ The LAB NMR measurements were performed using a Bruker 300 MHz Advance III Spectrometer, with an MRI probe and a custom flow-through NMR sampling tube.^16^

### BMS Theory

The BMS effect and hyperfine interactions experienced by a nucleus are additives, and the total shift caused by a paramagnetic species can be expressed as^13^
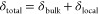
1where δ_local_ is the shift caused by local hyperfine interactions between the unpaired electrons of the paramagnetic species and the nuclei of interest.^13^ δ_local_ does not distinguish between the types of hyperfine interactions: Fermi contact or pseudo-contact. The δ_bulk_ is the shift caused by the BMS given by^17^
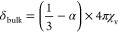
2where χ_v_ is the bulk volumetric magnetic susceptibility and α is the shape factor. This shape factor accounts for the effect of sample shape and orientation relative to the applied magnetic field (produced by the NMR magnet) on the magnetic field observed by the nuclei of interest. Non-isotropic macroscopic effects such as induced dipoles at the boundaries of the sample contribute to the shape factor.

The shape factor at a point within the sample,  can be calculated from the surface integral which gives the total magnetic flux through a point on the surface
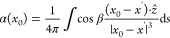
3where *x*^′^ is the location of the surface element, *ẑ* is the unit vector normal to the surface, and β is the angle between the applied magnetic field and the normal to the surface. For simple geometries, the shape factors are constant throughout the sample and have been explicitly solved: the shape factors for a sphere or infinite cylinder oriented either perpendicular or parallel to the applied magnetic field are summarized below.^17^
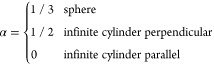
4

If we approximate our samples as infinite cylinders, we can see from eq 2 that the shift caused by a BMS effect for samples in a perpendicular orientation is δ_bulk_^⊥^ = −2/3πχ_v_ and that for samples in a parallel orientation, δ_bulk_^∥^ = 4/3πχ_v_. We can then write out eq 1 for a sample in either perpendicular or parallel orientations
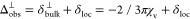
5
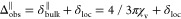
6where Δ_obs_^⊥^ and Δ_obs_^∥^ are the experimentally observed shifts caused by adding paramagnetic species into the solution; that is, Δ_obs_^⊥^ = δ_paramag_^⊥^ – δ_noparamag_^⊥^ and likewise, Δ_obs_^∥^ = δ_paramag_^∥^ – δ_noparamag_^∥^. Solving the system of equations allows the calculation of χ_v_ and δ_loc_ from experimentally measured values
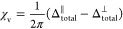
7
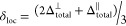
8

Thus, orientation-dependent measurements can be used to deconvolute the BMS and hyperfine effects caused by paramagnetic dissolved oxygen in LAB electrolytes. This method was described by Delpuech et al. to compare O_2_ solubility in fluorinated benzene with benzene^18^ and built upon earlier paramagnetic NMR studies first described by Evans and later by Becconsall et al., as well as Garroway.^14,19,20^

Once the volumetric magnetic susceptibility χ_v_ is determined, it is used to calculate the concentration of paramagnetic species^16^
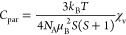
9where *C*_par_ is the concentration of paramagnetic species, *k*_B_ is the Boltzmann’s constant, *T* is the temperature, *N*_A_ is Avogadro’s number, μ_B_ is the Bohr magneton, and *S* is the total spin quantum number (*S* = 1/2 for each unpaired electron). For O_2_ with two unpaired electrons, *S* = 1, and solving for the constants, we end with
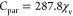
10

Equations 7 and 10 allow us to directly calculate the concentration of dissolved O_2_ in mM from our orientation-dependent NMR measurements.

## Results

### Oxygen Solubility—NMR Shifts and Relaxometry

**Figure 1:**
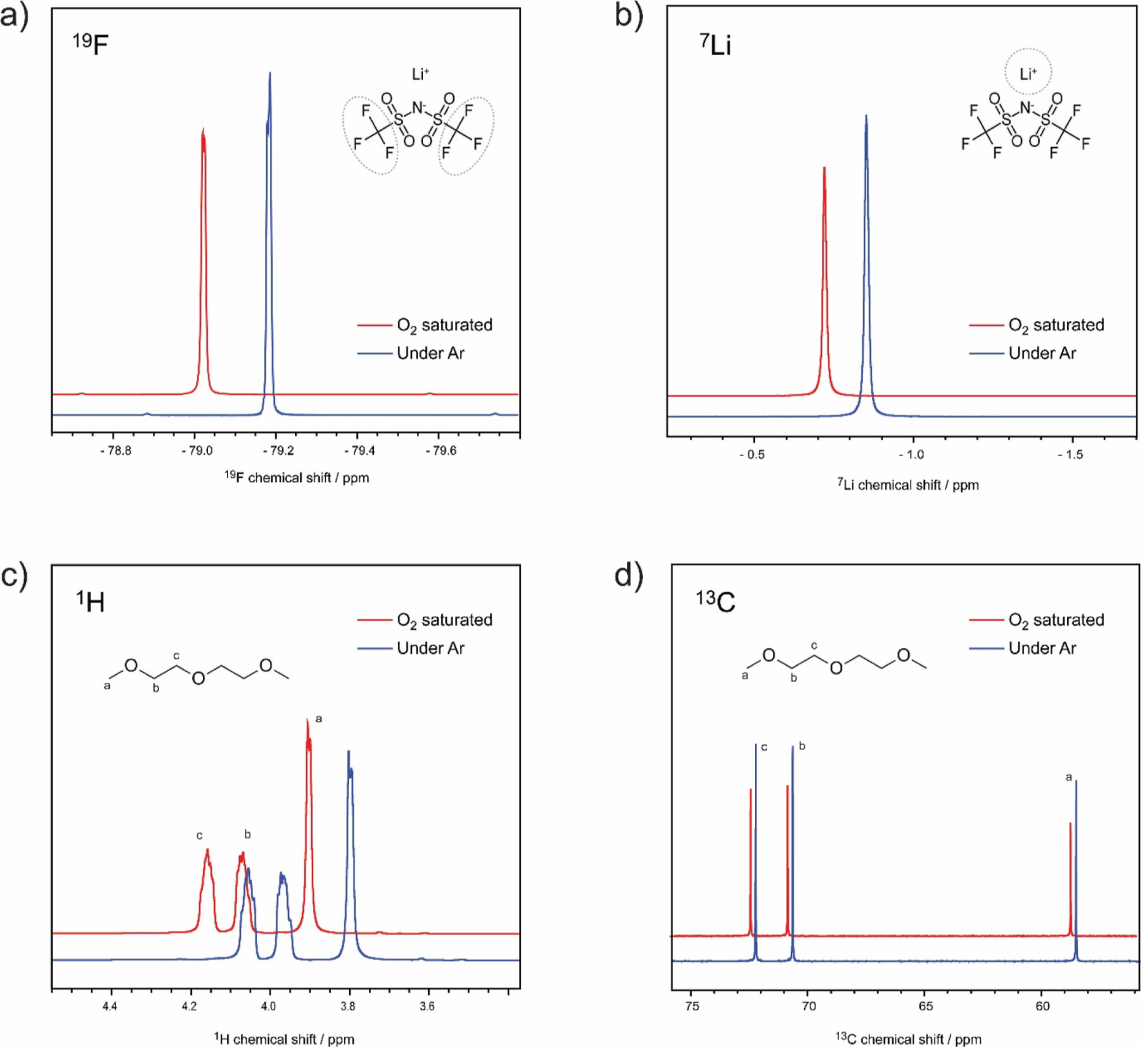
(a) ^19^F, (b) ^7^Li, (c) ^1^H, and (d) ^13^C solution NMR spectra of a 0.25 M LiTFSI in diglyme electrolyte before (blue) and after (red) saturation with O_2_, acquired in a standard, sealed NMR tube.

Figure 1 shows the ^19^F, ^7^Li, ^1^H, and ^13^C NMR spectra of a 0.25 M LiTFSI in diglyme electrolyte after it is prepared in the glovebox (blue trace) as well as after saturating the electrolyte with O_2_ (red trace). The ^19^F and ^7^Li resonances result from the TFSI^–^ salt anion and Li^+^ cation, respectively, while the ^1^H and ^13^C spectra result from the diglyme solvent. The measured NMR shifts caused by the addition of O_2_ into the LiTFSI-diglyme electrolyte were 0.156 ppm for ^19^F, 0.12 ppm for ^7^Li, 0.133 ppm for ^1^H, and 0.125 ppm for ^13^C. The shifts caused by O_2_ are not uniform across the different nuclei, suggesting that O_2_ does not interact with the nuclei present in the electrolyte solely through a bulk effect: there are also local interactions between the paramagnetic electrons on the O_2_ molecules and the nearby salt or solvent molecules, eq 1. The O_2_-induced shifts observed in either the ^13^C or ^1^H spectra are the same for the −CH_3_ and −CH_2_ peaks, within the resolution of this method.

**Figure 2:**
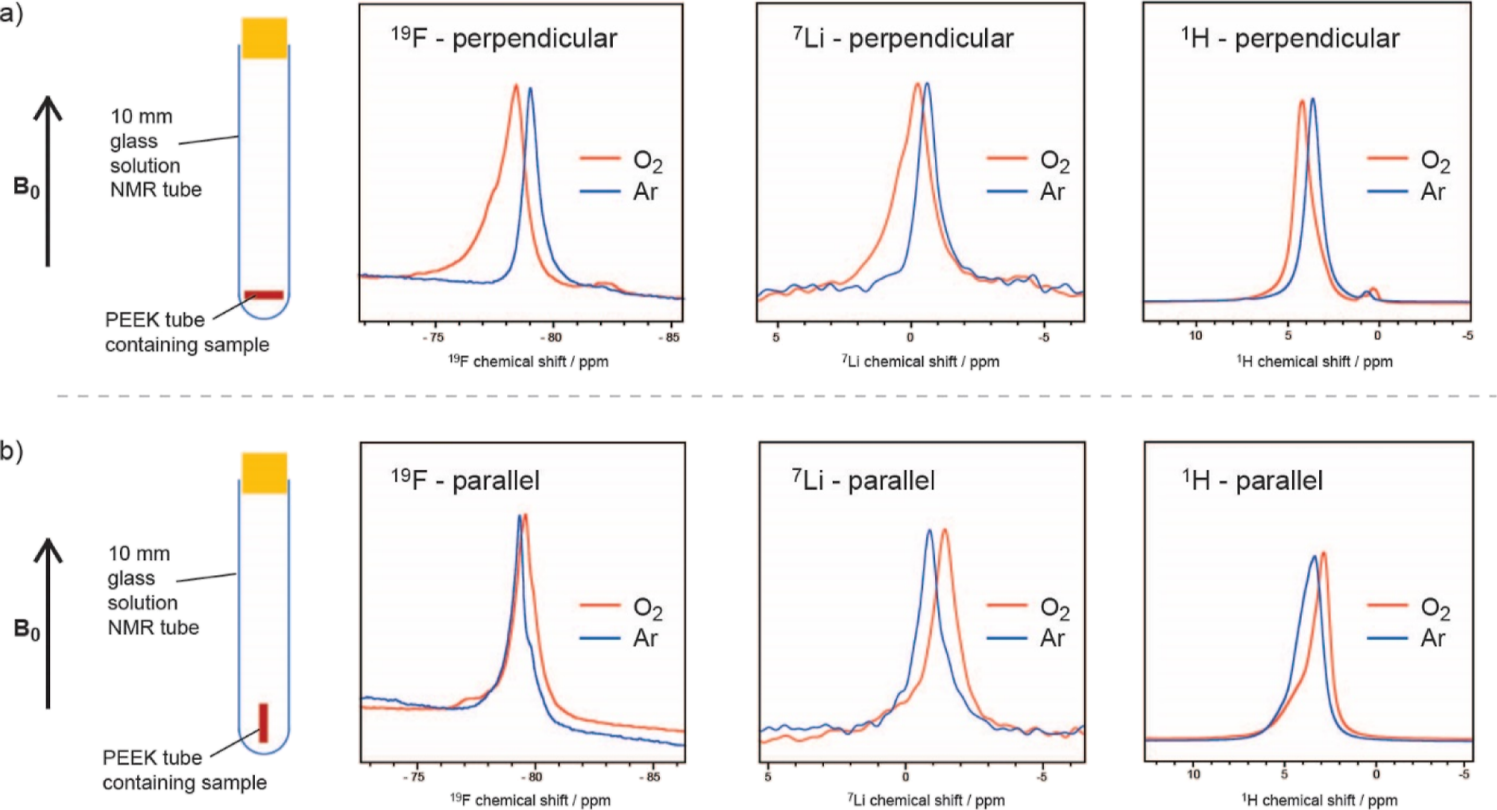
(a) ^19^F, ^7^Li, and ^1^H NMR spectra of the 0.25 M LiTFSI in diglyme electrolytes where the sample is oriented perpendicular to the applied magnetic field, before (blue) and after (orange) O_2_ saturation. The ^1^H NMR peak shown is the −CH_3_ peak of the diglyme solvent. (b) ^19^F, ^7^Li, and ^1^H NMR spectra of the same electrolyte where the sample is oriented parallel to the applied magnetic field.

In order to untangle the shift induced by a bulk effect from any local effects caused by the paramagnetic O_2_ molecules, solution NMR measurements were made with the samples oriented at two different angles to the external magnetic field (Figure 2). The electrolyte samples were first oriented perpendicular to the applied magnetic field as shown in the schematic in Figure 2a: a 7 mm PEEK tube was filled with the electrolyte (shown in dark red) and then positioned horizontally at the bottom of a 10 mm glass solution NMR tube. Figure 2b shows the NMR measurements for the electrolyte samples oriented parallel to the magnetic field, with the 7 mm PEEK tube positioned vertically into the glass tube. This positioning was possible due to static cling to the glass tube (see Supporting Information for an image of the sample). The electrolyte samples were measured with and without O_2_ saturation, and the ^19^F, ^7^Li, and ^1^H spectra for the 0.25 M LiTFSI in diglyme electrolyte are shown in Figure 2, where only the peaks corresponding to the −CH_3_ protons are shown in the ^1^H spectra. The spectra were referenced by setting the shift positions measured under Ar (without O_2_) to the values observed inside a standard NMR tube (i.e., Figure 1), which accounts for any diamagnetic contributions to the BMS. The measurements were then repeated for different electrolytes: 0.25 M LiTFSI in triglyme, tetraglyme, and DMSO. A monoglyme (also known as DME—dimethoxyethane)-based electrolyte was not measured due to its volatility since bubbling in O_2_ gas vaporizes the solvent and changes the LiTFSI concentration.

In order to calculate the volumetric susceptibility and the hyperfine shift, the observed change in shift caused by adding O_2_ was first calculated for the ^19^F, ^7^Li, and ^1^H (CH_3_−) NMR spectra by subtracting the NMR resonance of a chosen peak for the non-O_2_ electrolyte (Ar) from the NMR resonance of the O_2_-saturated electrolyte, defined for the perpendicular electrolyte sample as . Similar calculations were performed for the parallel orientation to extract Δ_obs_^∥^. Once Δ_obs_^⊥^ and Δ_obs_^∥^ were calculated, eqs 7 and 8 could be used to determine the shift caused by the BMS effect (and from that χ_v_) and the hyperfine shifts (δ_loc_) for ^19^F, ^7^Li, and ^1^H (Table 1). It is encouraging to note that the calculated volumetric magnetic susceptibilities were similar in value for the different nuclei, as expected for a bulk effect.

**Table 1:** Calculated Volumetric Susceptibility (χ_v_, Unitless) and Hyperfine Shift (ppm) from Orientation Measurements of Electrolytes before and after O_2_ Saturation^a^

	^19^F		^7^Li		^1^H	
solvent (0.25 M LiTFSI)	χ_v_	δ_loc_	χ_v_	δ_loc_	χ_v_	δ_loc_
diglyme	0.031 ± 0.001	–0.35 ± 0.01	0.030 ± 0.001	–0.01 ± 0.01	0.028 ± 0.005	–0.05 ± 0.02
triglyme	0.018 ± 0.003	–0.18 ± 0.01	0.017 ± 0.001	–0.01 ± 0.01	0.016 ± 0.005	–0.03 ± 0.01
tetraglyme	0.011 ± 0.005	–0.15 ± 0.01	0.011 ± 0.003	–0.02 ± 0.01	0.008 ± 0.007	+0.01 ± 0.02
DMSO	0.006 ± 0.001	–0.23 ± 0.01	0.007 ± 0.005	–0.11 ± 0.01	0.007 ± 0.006	–0.05 ± 0.01

a Errors included were calculated from the variance in the Δ_obs_^⊥^ and Δ_obs_^∥^ values from repeat measurements (each measurement was carried out twice).

Using the calculated χ_v_, we applied eq 10 to calculate the concentration of O_2_ for the different electrolytes and compared them to literature values (Table 2). For the electrolytes measured in this work, the calculated O_2_ concentration values are comparable to those previously reported and measured via different methods: Gittleson et al. used electrochemical methods,^9^ while Hartmann et al. and Schürmann et al. used pressure uptake measurements.^11,21^ For the diglyme-based electrolyte, increasing salt concentration also led to a decrease in O_2_ saturation. These trends are again consistent with those reported in the literature.^9^ The relatively large errors of the method reflect the difficulty in ensuring that the solution is saturated with O_2_ and errors in the orientations of the samples. We note that it would be straightforward to improve the method to reduce the errors by designing bespoke NMR cells.

**Table 2:** O_2_ Saturation Concentrations in mM from NMR Measurements (Calculated from χ_υ_ from Measurements of the Three Nuclei) and the Literature^a^

		this work: concentration of O_2_ (mM)					
solvent	LiTFSI concentration (M)	^19^F	^7^Li	^1^H	from ref (9)	from ref (21)	from ref (11)
diglyme	0.125	9.3 ± 0.4	8.9 ± 0.8	8.5 ± 0.4		6.4*	7.1*
	0.25	9.0 ± 0.4	8.7 ± 0.4	8.2 ± 0.4			
	0.5	8.7 ± 0.4	8.8 ± 0.9	6.2 ± 0.8			
	1	8.6 ± 1.4	8.3 ± 0.9	5.6 ± 1.4			
triglyme	0.25	5.3 ± 0.9	5.0 ± 0.4	4.7 ± 1.4		5.6*	5.6*
tetraglyme	0.25	3.3 ± 1.4	3.3 ± 0.8	2.3 ± 2.1	2.1**	4.3*	4.3*
DMSO	0.25	1.7 ± 0.4	1.9 ± 1.4	2.1 ± 2.0	0.99**		

a The experimental errors were again calculated from the variance in the Δ_obs_^⊥^ and Δ_obs_^∥^ values from repeat measurements (each measurement was carried out twice). * indicates literature values for the solvent only (no salt added). ** indicates a value for a 0.5 M LiTFSI salt concentration in the electrolyte.

**Figure 3:**
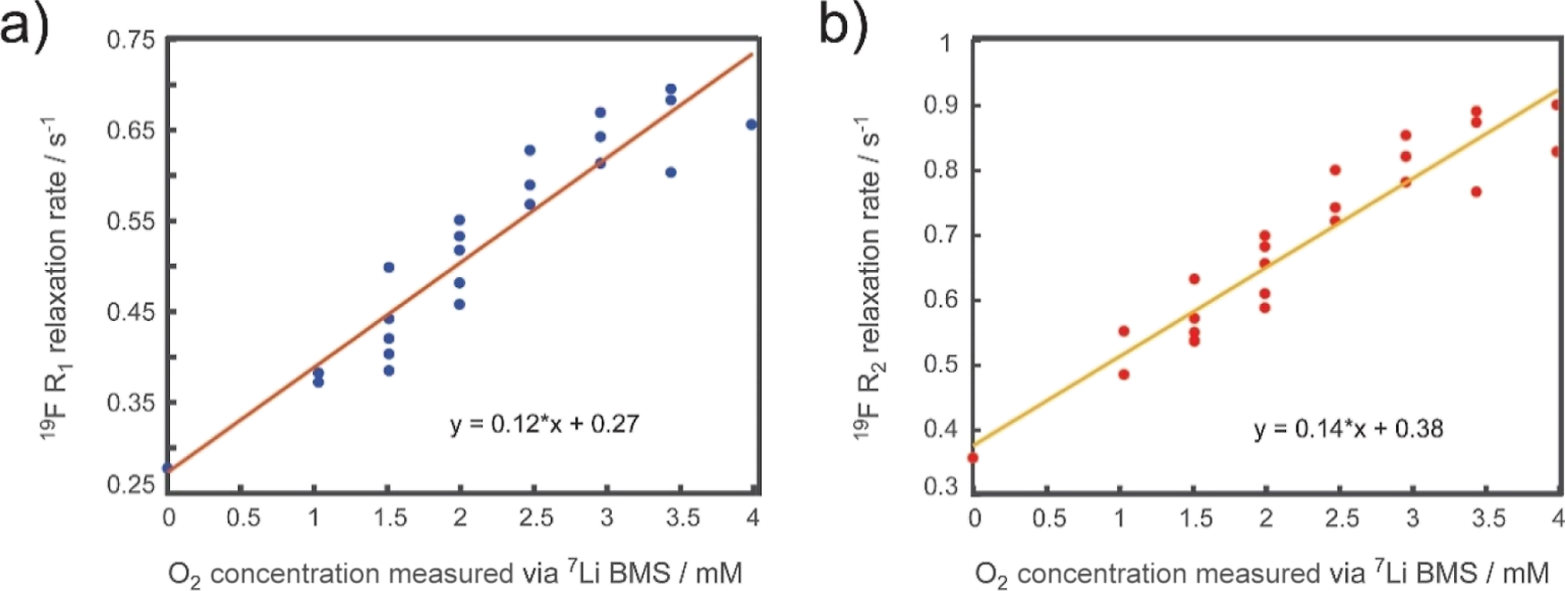
(a) ^19^F longitudinal and (b) transverse relaxation rates plotted as a function of O_2_ concentration in the 0.25 M LiTFSI in diglyme electrolyte, where O_2_ concentration was measured via ^7^Li BMS.

The longitudinal (*R*_1_) and transverse (*R*_2_) relaxation values were then measured as a function of O_2_ concentration for the 0.25 M LiTFSI in diglyme electrolyte, as plotted in Figure 3a,b, respectively. ^19^F was used for relaxation measurements due to its increased sensitivity to O_2_ concentration compared to other nuclei (see Figure S3). The observed ^7^Li shift was then directly used to calculate O_2_ concentrations using eqs 6 and 10 since the hyperfine shift for this nucleus is essentially zero (see Table 1). Thus, relaxation rates can be compared with O_2_ concentrations. A linear behavior with respect to oxygen concentration was observed, allowing a calibration curve for estimating O_2_ concentration in the electrolyte from measured relaxation rates to be obtained. This method of using sensitive ^19^F relaxometry to quantify O_2_ has previously been used in biological systems.^22^

### Oxygen Diffusivity

**Figure 4:**
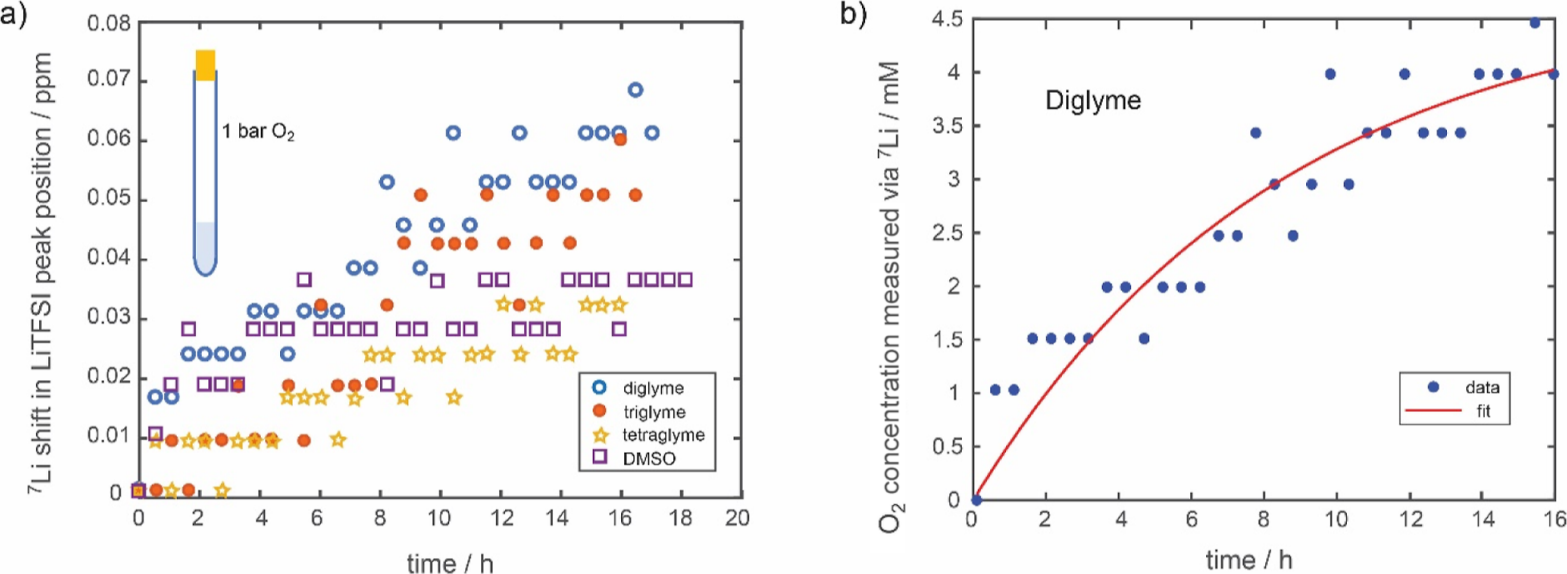
(a) Change in ^7^Li NMR LiTFSI peak position as a function of time for various electrolytes. The gas overhead space in a solution NMR tube was filled with O_2_ gas. For each sample, 0.3 mL of electrolyte was used, (b) exponential fitting of the O_2_ concentration versus time for the diglyme electrolyte as measured from the change in ^7^Li chemical shift.

We then designed experiments to measure oxygen diffusivity via NMR: rather than saturating the electrolyte with O_2_, the gas overhead space in the NMR tube was filled with O_2_, Figure 4a inset. In this way, O_2_ from the gas phase needs to dissolve into the electrolyte, and the subsequent change in NMR chemical shift is measured as a function of time. Figure 4a shows the change in ^7^Li chemical shift caused by O_2_ diffusion into the electrolyte for the different electrolytes. Relatively small changes in the chemical shifts are observed in the ^7^Li spectra, on the order of only 0.01–0.02 ppm per time step. These small changes, in conjunction with possible errors such as minor temperature fluctuations, gave rise to the noise observed in Figure 4. Nonetheless, an increasing ^7^Li shift as a function of time caused by the dissolution of paramagnetic O_2_ is clearly observed. Furthermore, an increase in O_2_ dissolution/diffusion rate with decreasing molecular weight glymes is consistent with literature reports and increasing viscosity effects. Plotting the ^19^F shift in LiTFSI peak position as a function of time for the same experimental setup results in a larger shift resolution (Figure S2); however, the ^19^F peak shifts include hyperfine contributions that are difficult to deconvolute in the experimental setup.

The diffusion data was fit using an exponential growth function. Fitting oxygen dissolution data is well studied in the bio-reactor literature^23^
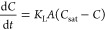
11where eq 11 can be integrated to give
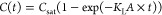
12

*C*(*t*) is the concentration of O_2_ as a function of time, C_sat_ is the saturated oxygen concentration, *K*_L_ is related to the oxygen transfer coefficient (from the gas phase to dissolving into the solvent), which is directly proportional to diffusivity, and *A* is the surface area to volume ratio of the liquid. In two film theories, it is assumed that molecular diffusion is the only mechanism of O_2_ transfer, and therefore, *K*_L_ is equal to the diffusion coefficient divided by the distance over which the O_2_ needs to dissolve.^23^ Knowing the geometric parameters (volume to surface area ratio and diffusion distance), *K*_L_ and diffusivity can be extracted from the fitting (see Supporting Information for fitting other electrolytes). Table 3 lists the calculated O_2_ diffusivity from the ^7^Li NMR diffusion experiments and compares them with literature values.

**Table 3:** Calculated O_2_ Diffusion Coefficients from Fitting Chemical Shift Data^a^

solvent	LiTFSI concentration (M)	this work: diffusivity of O_2_(cm s^–1^)	from ref (9) (cm s^–1^)	from ref (21) (cm s^–1^)	from ref (11) (cm s^–1^)
diglyme	0.25	7.8 × 10^–5^± 2.9 × 10^–5^		4.4 × 10^–5^*	4.6 × 10^–5^*
triglyme	0.25	4.8 × 10^–5^± 2.6 × 10^–5^		3.2 × 10^–5^*	3.5 × 10^–5^*
tetraglyme	0.25	4.6 × 10^–5^± 2.0 × 10^–5^	3.97 × 10^–7^**	2.6 × 10^–5^*	2.6 × 10^–5^*
DMSO	0.25	4.0 × 10^–5^± 1.2 × 10^–5^	1.40 × 10^–5^**		

a Errors listed in the table for the diffusivity values calculated in this work indicate the 95% confidence interval calculated for the parameter fitting. * indicates a measurement for the solvent only (no salt added). ** indicates a measurement for a 0.5 M LiTFSI electrolyte.

### LAB Cells

**Figure 5:**
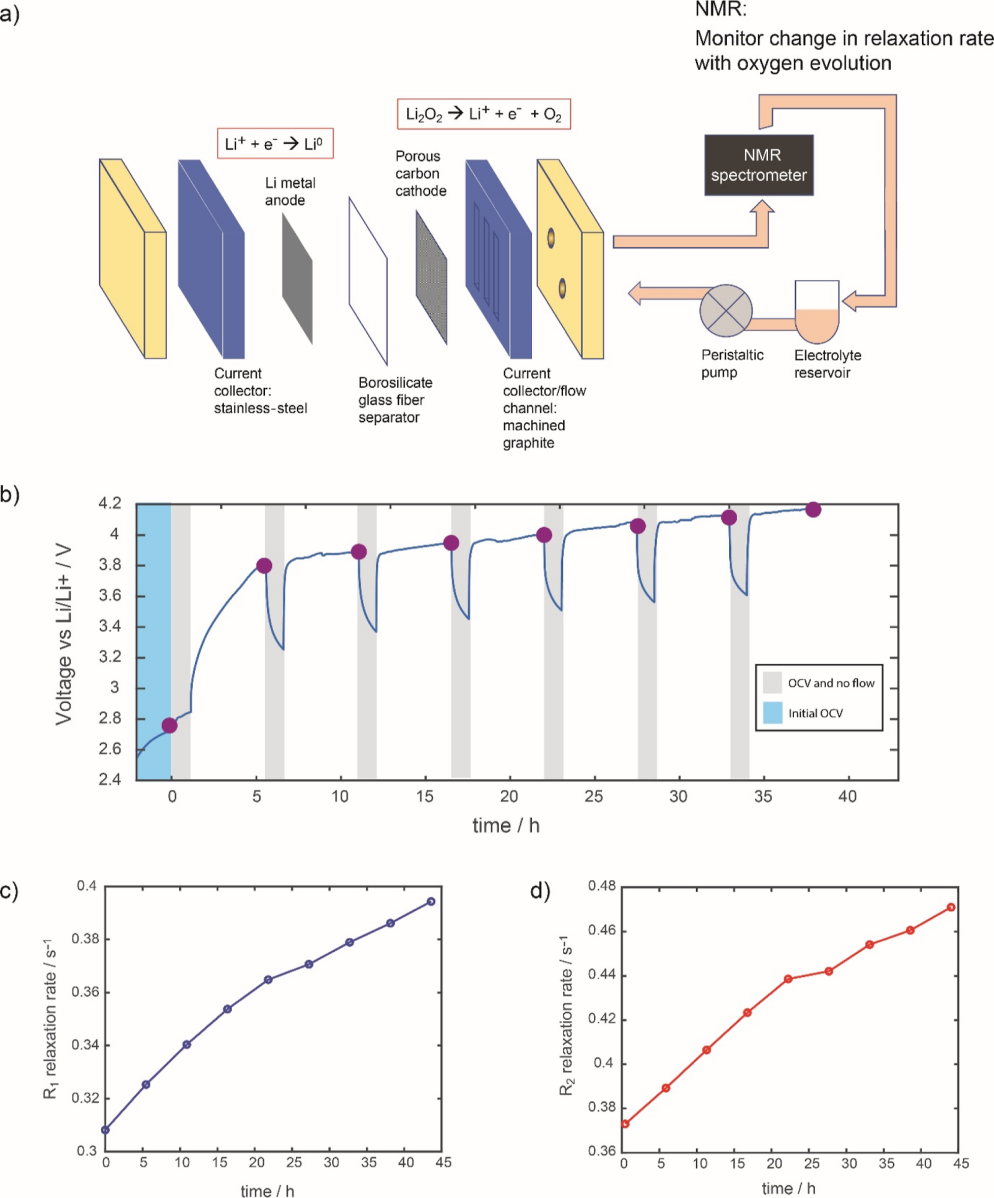
(a) Schematic of the Li–air flow battery during charge with electrolyte flowing through the NMR spectrometer. (b) Voltage versus time profile for the Li–air flow battery under galvanostatic charge. Every 4 h, the current and pump are paused, as highlighted in the gray regions. The purple circles indicate the points when relaxometry measurements were taken. The final 4 h charge and OCV were not included in this plot. (c) Longitudinal, *R*_1_, and (d) transverse, *R*_2_, relaxation rates measured during charge.

The effects of O_2_ evolution on NMR parameters in a working LAB device were then explored. A flow-cell design setup with in situ NMR measurements was used where the electrolyte flows from the cell into the spectrometer and then back into the reservoir during cell operation to monitor dissolved O_2_ concentrations (Figure 5a). The cell was charged under galvanostatic conditions to oxidize the Li_2_O_2_ formed during discharge, and O_2_ was detected via NMR relaxometry measurements. Relaxometry methods were used rather than measuring changes in the chemical shifts due to the small amounts of O_2_ evolved during charge; changes in the chemical shifts of the ^7^Li spectra would be very small and difficult—although not impossible—to measure accurately.

Figure 5b shows the voltage profile during charge for the Li–O_2_ battery. The galvanostatic charge was paused every 4 h during relaxation measurements, seen as the drops in voltage down to open-circuit potential values. During the pause (relaxation measurements took an hour in total), the pump was also paused to halt the electrolyte flow. The large overpotentials observed during charge and drops during pauses are typical of a LAB without redox mediators. Figure 5c,d shows the measured *R*_1_ and *R*_2_ rates, respectively, collected every 4 h during charge. These rates increase with increasing state of charge, as expected due to increased paramagnetic O_2_ concentration in the electrolyte. Using the calibration curves from Figure 4, the O_2_ concentrations can be calculated from the relaxation rates; although the value of *R*_1_ measured before charging is larger than observed in the ex situ electrolyte measurements, the change in O_2_ concentration measured via *R*_1_ and *R*_2_ is similar (Table 4).

**Table 4:** Summary of the LAB Charge (from Figure 5) and O_2_ Evolution as Calculated from Capacity or Relaxometry Measurements

time (h)	charge passed (mA h)	*R*_1_ rate measured (s^–1^)	*R*_2_ rate measured (s^–1^)	[O_2_] from charge (mM)	[O_2_] from *R*_1_ rate (mM)	[O_2_] from *R*_2_ rate (mM)
0	0	0.31	0.37	0	0.27	0
5.5	0.22	0.32	0.39	0.16	0.45	0.20
11	0.44	0.34	0.41	0.33	0.55	0.30
16.5	0.66	0.35	0.42	0.49	0.70	0.50
22	0.88	0.37	0.44	0.66	1.0	0.65
27.5	1.1	0.37	0.44	0.82	1.05	0.70
33	1.3	0.38	0.45	0.97	1.1	0.80
38.5	1.5	0.385	0.46	1.1	1.15	0.90
44	1.8	0.39	0.47	1.3	1.2	1.0

These O_2_ concentrations are compared with the theoretical O_2_ evolution calculated from the charge passed (assuming 100% coulombic efficiency) in Table 4, with the concentrations calculated via relaxometry measurements being lower than the expected theoretical O_2_, calculated assuming 100% coulombic efficiency. Discrepancies in the measured O_2_ and the expected concentration of O_2_ evolved can be accounted for by parasitic reactions that consume electrons rather than oxidizing O_2_.^39,40^Figure S5 similarly shows poor coulombic efficiency of O_2_ evolution as measured via pressure, whereby not all of the O_2_ consumed on discharge was recovered on charge. Figure S6 shows the decreased capacity for the subsequent discharge and charge cycle of the LAB cell shown in Figure 5; the decreased capacity in Figure S6 is again indicative of discharge products (desired or parasitic) that were not removed upon charge in the first cycle and can therefore help explain the lack of O_2_ evolution.

## Discussion

The oxygen diffusivities and solubilities in the electrolytes used, as determined via NMR in this work, were comparable to values previously recorded in the literature. This suggests that measuring oxygen concentrations using the chemical shift induced by the BMS effect is an effective method for estimating concentrations. Across the measurements, the NMR peaks broadened when O_2_ was introduced because of increased relaxation rates.^13^ The relaxometry measurements plotted in Figure 3a,b also demonstrate an increase in relaxation rates with increasing O_2_ concentration. This is expected as the relaxation rate is proportional to the spatial distance between paramagnetic species and the nuclei of interest.

General O_2_ solubility and diffusivity trends in the glyme series are reproduced with this method, where the decrease in O_2_ diffusivity with increasing molecular weight of the glyme solvent, going from diglyme to triglyme and tetraglyme, is likely a viscosity effect as described by the Stokes–Einstein equation.^9^ Possible explanations for variations in solubilities and diffusivities between our measurements and literature values may be a result of approximating sample geometries as infinite cylinders, as well as the differences in concentrations of electrolyte salts used or differences in O_2_ saturation (i.e., our samples or theirs may not have been fully saturated due to loss of O_2_ on transfer between tubes), as well as possible temperature effects.

There appears to be an overestimation in value for the diffusion measured via NMR compared to reported literature values;^9,11,21^ although oxygen is known to have higher solubilities and diffusivities in nonaqueous solvents compared to aqueous solvents,^24,25^ the oxygen diffusion value measured here for diglyme electrolyte approaches the diffusion coefficient of protons in water.^26^ Potential causes for the overestimation in diffusion coefficients may be a result of the large diffusion length used in the calculation for *K*_L_ and any inaccuracies in fitting caused by the resolution limits of the NMR spectra when measuring small shifts in peak positions caused by dissolving oxygen.

In addition to providing matches to previously measured O_2_ solubilities and diffusivities, the NMR methodology also reveals information on the O_2_ solvation environments within the electrolytes. As observed in the chemical shifts and calculated local hyperfine shifts of different nuclei (Table 1), O_2_ appears to interact or situate more closely, on average, with F atoms in LiTFSI-glyme electrolytes than it does with Li^+^ ions or the glyme protons. There is also a larger effect of O_2_ concentration on relaxation rates in ^19^F compared to other nuclei, as observed via the larger slope calculated when plotting relaxation rates as a function of O_2_ concentration for ^19^F (see Figure S3). The F atoms may be more accessible to O_2_ than Li^+^ ions in the glyme electrolytes studied since glymes are known to solvate Li^+^ ions, forming chelation complexes,^27,28^ and making it more difficult for O_2_ to bind to Li^+^.

In contrast, in the LiTFSI-DMSO electrolyte case, significant hyperfine shifts were seen for both ^19^F and ^7^Li (−0.23 and −0.11 ppm, respectively), for a saturated concentration of O_2_ of approximately 2 mM (Table 1). These compare to hyperfine shifts of −0.15 and −0.02 ppm for ^19^F and ^7^Li, respectively, observed for tetraglyme, despite tetraglyme having a higher concentration of dissolved O_2_ (3–8 mM, Table 1). DMSO does not chelate effectively with the Li^+^ ions,^10^ which may result in increased O_2_–Li^+^ binding, resulting in larger hyperfine interactions and thus ^7^Li hyperfine shifts (as compared to glymes).

Paramagnetic oxygen has been used to probe, for example, cation-binding sites in molecular sieves.^29−32^ Previous literature using ^6,7^Li and ^133^Cs NMR have shown large positive shifts for cations that are able to bind directly to O_2_.^29−31^ These observed positive shifts are caused by Fermi-contact or through-bond interactions.^30^ By contrast, a small negative shift was observed for protons in Brønsted acid sites of zeolites coordination to O_2_.^32^ While further calculations are required, the negative shifts seen here for Li^+^ in DMSO-containing electrolytes suggest that a dipolar (pseudo-contact) mechanism may be important in the coordination of dissolved O_2_ to Li^+^ in these electrolytes, with dipolar coupling being inversely proportional to the third power of the distance (between O_2_ and Li^+^, in our case). Similarly, a pseudo-contact mechanism likely plays a role in O_2_ to TFSI binding.

MD simulations were performed on an O_2_-saturated electrolyte of 0.25 M LiTFSI in diglyme to further verify the O_2_ solvation environment observed via the NMR. Closer F–O_2_ as compared to Li^+^–O_2_ interactions are seen, where the extracted radial distribution functions show Li^+^ density at distances of greater than 5 Å from O_2_ (Figure S4). Our simulations also suggest an increase in the normalized density of F atoms around O_2_ compared to the H atoms around dissolved O_2_ (Figure S4). These agree with MD simulations performed by Haas et al., who observed more TFSI^–^ anions surrounding dissolved O_2_ than solvent molecules.^10^

Our NMR observations also align with previous work by Hamzah et al., where they observed O_2_ affinity for F atoms when using ^1^H and ^19^F NMR to probe dissolved oxygen in benzene vs fluorinated benzene.^33^ The paramagnetic effects of O_2_ on ^19^F NMR spectra and the oxyphilic nature of F atoms have also been well studied, and the approach is often used in biological samples.^22,34,35^ For example, Taylor et al. used ^19^F relaxometry to study oxygen uptake in a suspension of bacterial cells.^34^ Furthermore, fluorinated solvents are a class of artificial oxygen carriers and can be used in emulsions for blood replacement due to their high O_2_ solubilities.^25^ As such, increasing fluorination of LAB electrolytes has been proposed as an approach to improve O_2_ transport.^36−38^ While some work has suggested that fluorinated compounds can be used as additives or as co-solvents, the challenges of low Li salt solubility and Li^+^ ion conduction in fluorinated compounds remain to be solved. The NMR methodology in this work could be applied to these systems to characterize the hyperfine interactions between different nuclei with O_2_, to better understand how O_2_ is solvated, and to correlate solvation modes with solubility limits.

We also used our NMR methodology to perform operando measurements to quantify evolved O_2_ during cell operation. Discrepancies in the measured O_2_ and the expected concentration of evolved O_2_ can be accounted for by parasitic reactions that consume electrons rather than generating O_2_:^39,40^ the evolution of O_2_ from Li_2_O_2_ oxidation does not occur at a constant rate, nor at 100% faradaic efficiency, as shown using differential electrochemical mass spectroscopy, suggesting parasitic reactions consuming electrons, for instance, to form/oxidize Li_2_CO_3_.^41^ In Figure S5, we calculated the ratio of the moles of electrons consumed on charge (from the current) to the moles of O_2_ determined via pressure measurements: 2.8 moles of e^–^ were consumed per mole of O_2_ evolved, deviating from the ideal ratio of 2 moles of e^–^ per mol of O_2_ (Li_2_O_2_ → 2Li^+^ + 2e^–^ + O_2(gas)_). Calculating the ratio of moles of electrons consumed on charge to moles of O_2_ measured via NMR relaxometry for the LAB flow cell (Figure 5 and Table 4) gives a ratio of 2.2 moles e^–^ per mole of evolved O_2_ (see Supporting Information for calculation). Thus, the flow cell demonstrates an improved performance compared to the static LAB, which we ascribe to the improved mass transport of dissolved O_2_.^4^

Further explanations for the difference between measured and expected O_2_ concentrations may result from the chemical shift resolution limits when using the BMS effect in NMR and the errors in fitting the calibration curve. Oxygen may have also been released from the electrolyte and entered the gas overhead space in the electrolyte reservoir rather than remaining dissolved. Although more work is needed to understand the origin of some of the discrepancies, this experiment serves as a proof of concept for an alternative method to measure operando O_2_ evolution in a LAB electrolyte. Future work exploring MRI techniques using this method could potentially map out distributions in O_2_ concentrations within Li–O_2_ battery electrodes, which could feed into improved electrode design.

## Conclusions

In conclusion, this work presents a new methodology to measure dissolved O_2_ in LAB electrolytes via either the BMS shift or change in relaxation (*T*_1_ or *T*_2_) time of the observed nuclei. The measured solubilities were comparable to previously reported values in the literature, while the diffusivity values we measured were slightly overestimated but in the correct order of magnitude. The shifts induced by hyperfine interactions were separated from the BMS shifts by measuring samples aligned at different orientations to the field; the hyperfine shifts revealed preferential solvation of the dissolved O_2_ by F atoms in the TFSI^–^ salt anion, particularly in glyme electrolytes. To the best of our knowledge, experimental results showing O_2_ solvation in LAB electrolytes have not been reported prior to this work. Of the electrolytes measured, we observed the highest O_2_ solubility and diffusivity when using the lower molecular weight glyme as a solvent, as well as the largest shifts induced by hyperfine interactions with the F atoms, indicating that the O_2_ molecules spend more time, on average, near these atoms. Although DMSO-based electrolytes had lower O_2_ solubilities, large hyperfine interactions were observed for both ^19^F and ^7^Li, indicating that O_2_ interacts with both the Li^+^ ions and F atoms of the TFSI anions, suggesting competitive binding interactions and possibly also more TFSI^–^ in the Li^+^ coordination shell. Characterizing the O_2_ solubilities, diffusivities, and solvation environments in electrolytes is important in designing electrolytes for increased LAB capacities and rate capabilities. Finally, relaxometry methods were also used to quantify dissolved O_2_ during LAB cell operation using in situ measurements, where we observed limited coulombic efficiency. Our NMR method could therefore be applied to monitor O_2_ evolution in LAB systems with better coulombic efficiencies, such as with redox mediator additives, and validate the improvements.

## Supporting Information Available

Additional NMR spectra, calculations, and MD simulation details (PDF)

## Abbreviations

LAB: lithium–air battery
NMR: nuclear magnetic resonance
LiTFSI: lithium bis(trifluoromethane) sulfonylimide
BMS: bulk magnetic susceptibility
MD: molecular dynamics
RRDE: rotating ring-disk electrode
CPMG: Carr–Purcell–Meiboom–Gill
GF: glass fiber
PEEK: polyether ether ketone
PFA: perfluoroalkoxy alkanes
