# Supplementary material for: NMR Methodology for Measuring Dissolved O2 and Transport in Lithium–Air Batteries
Source: J Phys Chem C Nanomater Interfaces. 2023 May 22;127(21):10001–11. doi: 10.1021/acs.jpcc.3c00991 (PMC10240530; doi:10.1021/acs.jpcc.3c00991)
Supplement: Supplementary file 1 — jp3c00991_si_001.pdf [file jp3c00991_si_001.pdf]

# An NMR Methodology for Measuring Dissolved $O_2$ and Transport in Li-air Batteries (Supplemental Information)

*AUTHOR NAMES*

*Evelyna Wang[#], Erlendur Jónsson, Clare P Grey**

AUTHOR ADDRESS

Yusuf Hamied Department of Chemistry, University of Cambridge, Lensfield Road, Cambridge, CB2 1EW, UK

[#]Present Address: Chemical Sciences and Engineering Division, Argonne National Laboratory, Lemont, Illinois 60439, United States.

Figure S1: Photograph of the sample prepared for parallel alignment with the applied magnetic field. The 7 mm PEEK tube was inserted into the 10 mm glass NMR tube and stuck to the glass walls.

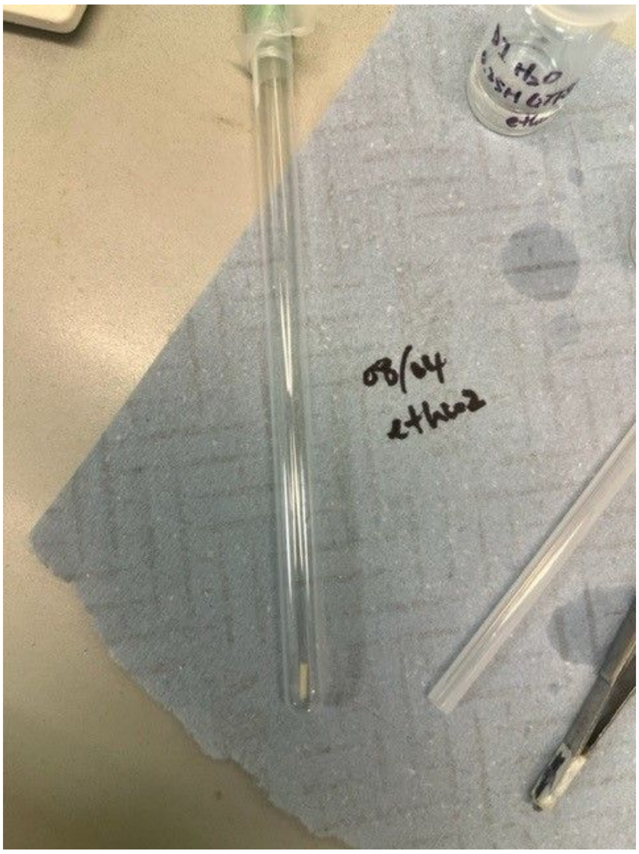

Figure S2: Dissolution experiment plotting the change in 19F shift as a function of time due to $O_2$ dissolution into the electrolyte (0.25M LiTFSI in diglyme). Error bars represent the standard deviation from average after 3 repeated experiments.

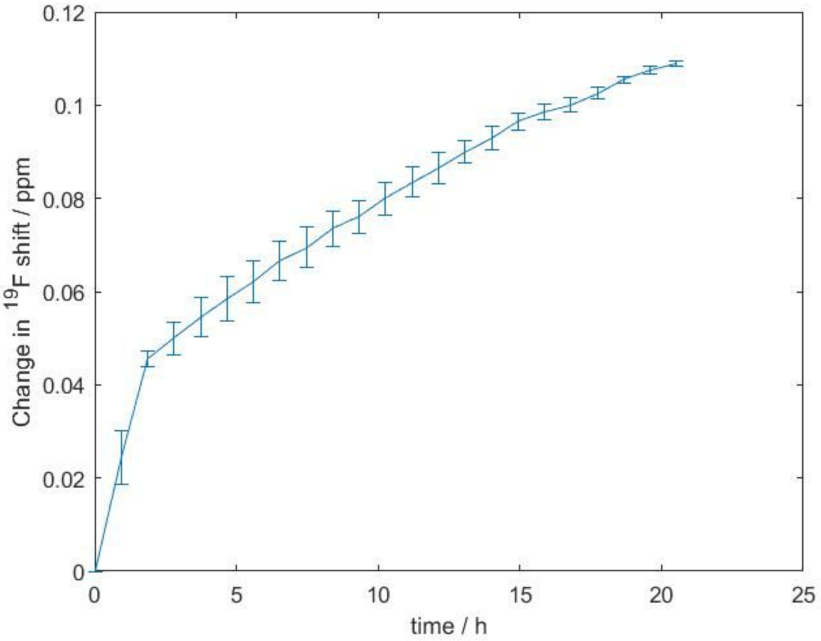

Figure S2: $O_2$ dissolution experiment and fittings for 0.25M LiTFSI in tetraglyme and triglyme using Equation 11 from the main text, $C(t) = C_{sat}(1 - \exp(-K_L A * t))$, where $C(t)$ is the concentration of $O_2$ as a function of time, $C_{sat}$ is the saturated oxygen concentration, $K_L$ is directly proportional to diffusivity, and $A$ is the surface area to volume ratio of the liquid.

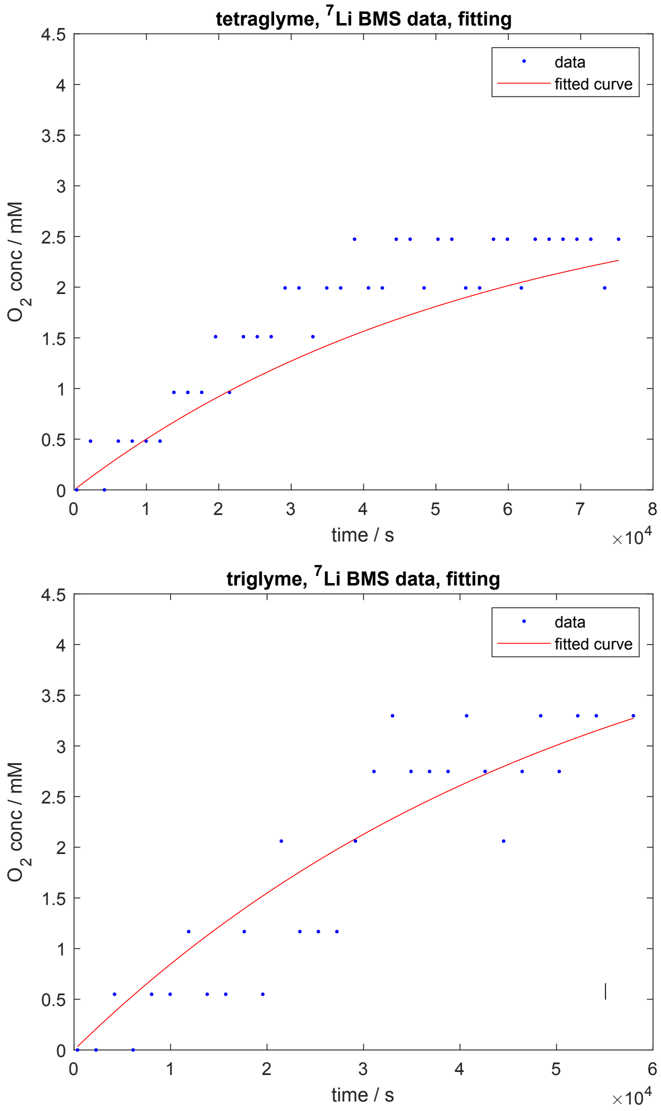

Figure S3: $^1$H relaxometry measurements at different dissolved $O_2$ concentrations for the 0.25M LiTFSI in diglyme electrolyte.

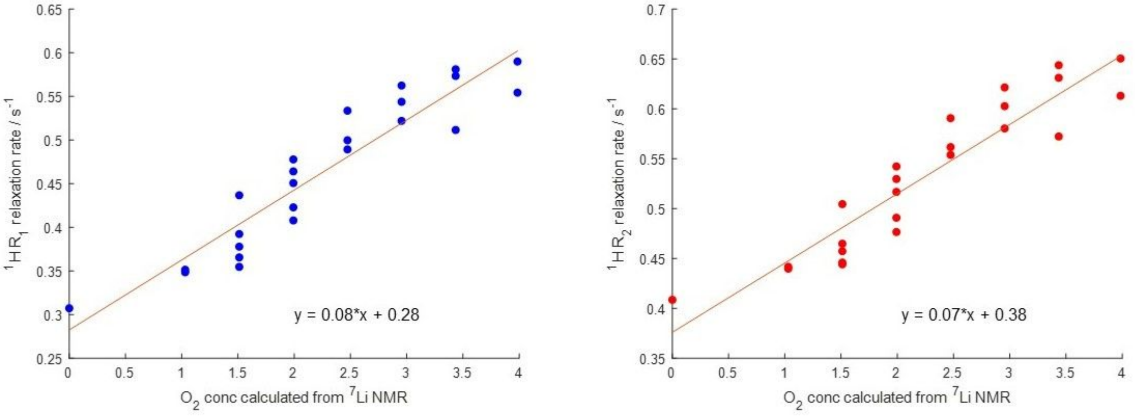

Details of molecular dynamics simulations:

The molecular dynamics (MD) simulation took the experimental concentrations of $O_2$ and LiTFSI in diglyme and used them as the basis for creating a simulation box, using Packmol.[1] The number of molecules were 1 $O_2$, 36 $Li^+$, 36 TFSI and 1000 diglyme. This box was then minimised with Gromacs version 2022.4,[2] which was used for the rest of the simulations.

The OPSL-AA force field was used for the diglyme (C-H bonds used constraints), while the TFSI used the parameters from Canongia Lopes and Pádua.[3] The molecular oxygen used the parameters from Arora and Sandler.[4] This particular combination of force fields has been tested previously and found to work well by Haas et al.[5]

Following the minimisation, an initial 100 ps NVT run was run with a Nosé-Hoover thermostat set to 298K with a 1 ps time constant. This was followed with a 5 ns NPT equilibration run, where the Parrinello-Rahman barostat was set to 1.01325 bar (1 atm) and a time constant of 5 ps and a compressibility of 4.5e-5. For the final NPT production run, the simulation was run for 10 ns, with data being stored every 2500 step. The simulations used a time step of 1 fs, throughout. Van der Waals and Coulombic interactions were cutoff at 1.2 nm. Following the simulation the built-in Gromacs toolchain, e.g. gmx rdf, was used to explore the structure of the electrolyte.

Figure S4: Radial distribution functions for F atoms, H atoms, and $Li^+$ relative to the $O_2$ molecule (centre of mass). No coordination of the $O_2$ to the $Li^+$ is observed.

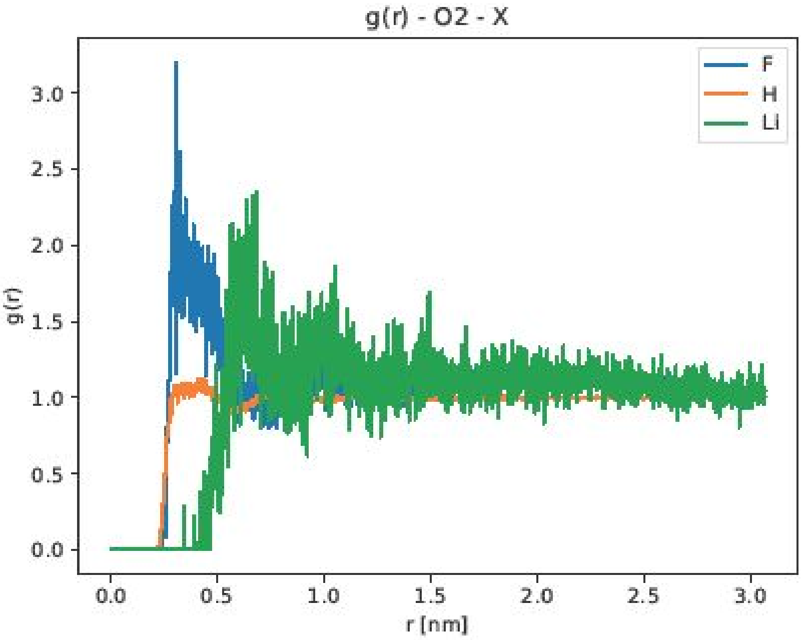

Figure S5 Discharge/charge voltage profile and operando pressure measurement for a LAB (Swagelok cell). The cell was cycled at 0.1 mA/cm$^2$ using a 0.25M LiTFSI in diglyme electrolyte, Li metal anode, and mesoporous carbon coated carbon paper. More electrons are consumed on charge than gas is evolved compared to the ideal ratio of 2 mol $e^-$ to 1 mol $O_2$ gas ($Li_2O_2 \rightarrow 2Li^+ + 2e^- + O_{2\,(gas)}$): 2.0 mole of electrons were consumed per mole of $O_2$ consumed on discharge and 2.8 mole of electrons were consumed per mole $O_2$ evolved on charge.

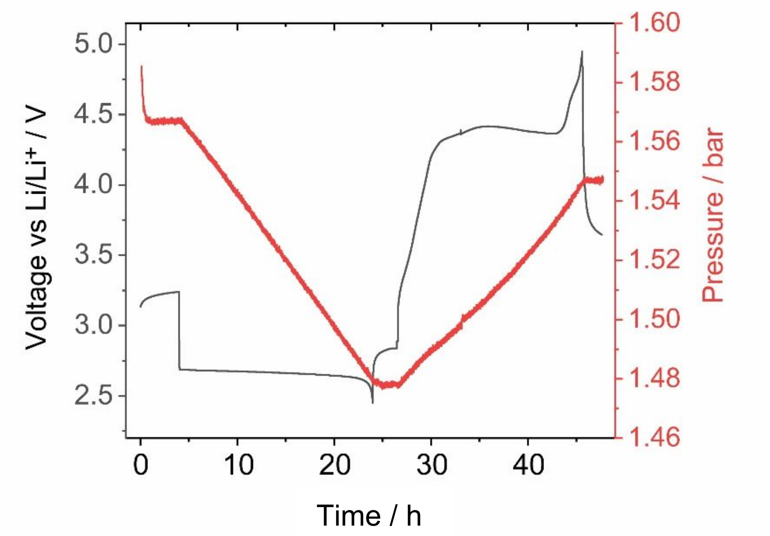

Figure S6 Subsequent discharge/charge voltage profile for the LAB cell shown in Figure 5, main text.

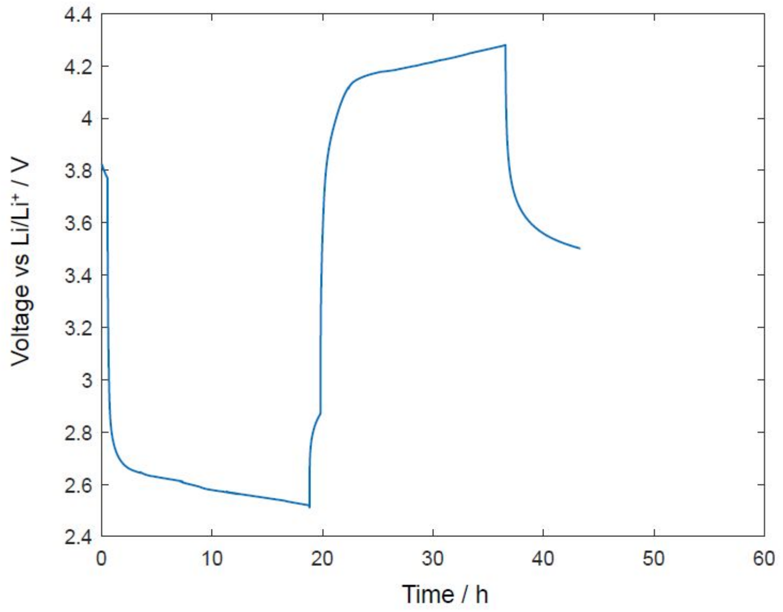

Calculation of the ratio of moles e⁻ to moles $O_2$ evolved from the operando NMR relaxometry experiment (Figure 5 and Table 4, main text)

Moles of electrons consumed measured via total charged passed:

$$1.8\ \text{mAh} = 1.8\ \text{mAh} * \frac{1\ \text{A}}{1000\ \text{mA}} * \frac{3600\ \text{s}}{\text{h}} * \frac{1\ \text{mole e}^-}{96485\ \text{C}} = 6.7 \times 10^{-5}\ \text{mole e}^-$$

Moles of $O_2$ measured via NMR relaxometry:

$$1.2\ \text{mM}\ O_2 = 1.2\ \text{mM}\ O_2 * \frac{1\ \text{M}}{1000\ \text{mM}} * 0.025\ \text{L} = 3 \times 10^{-5}\ \text{mole}\ O_2$$

Ratio of moles $e^-$ to moles $O_2$:

$$\frac{6.7}{3} = 2.2$$

**References**:


1. Martínez, L., et al. "Software news and update packmol: a package for building initial configurations for molecular dynamics simulations." *J. Comput. Chem* 30.13 (2009): 2157-2164. https://onlinelibrary.wiley.com/doi/10.1002/jcc.21224
2. Abraham, Mark James, et al. "GROMACS: High performance molecular simulations through multi-level parallelism from laptops to supercomputers." *SoftwareX* 1 (2015): 19-25. https://doi.org/10.1016/j.softx.2015.06.001
3. Canongia Lopes, José N., and Pádua, Agílio AH. "Molecular force field for ionic liquids composed of triflate or bistriflylimide anions." *The Journal of Physical Chemistry B* 108.43 (2004): 16893-16898. https://pubs.acs.org/doi/10.1021/jp0476545
4. Arora, Gaurav, and Sandler, Stanley I.. "Mass transport of $O_2$ and $N_2$ in nanoporous carbon ($C_{168}$ schwarzite) using a quantum mechanical force field and molecular dynamics simulations." *Langmuir* 22.10 (2006): 4620-4628. http://dx.doi.org/10.1021/la053062h
5. Haas, Ronja, et al. "Understanding the transport of atmospheric gases in liquid electrolytes for lithium–air batteries." *Journal of The Electrochemical Society* 168.7 (2021): 070504. http://dx.doi.org/10.1149/1945-7111/ac0d66
